# Peritonitis from injury of an aberrant subvesical bile duct during laparoscopic cholecystectomy: A rare case report

**DOI:** 10.1002/ccr3.1692

**Published:** 2018-07-09

**Authors:** Sanghyun Song, Sungho Jo

**Affiliations:** ^1^ Department of Surgery Dankook University Hospital Dankook University College of Medicine Cheonan‐si Chungnam Korea

**Keywords:** bile duct, cholangiopancreatography, cholecystectomy, leak

## Abstract

Aberrant subvesical bile ducts are rare anatomical structures. Damage to these ducts leads to bile leakage and can result in life‐threatening complications. Surgeons should be cautious that such a structure may be present, and surgery should be performed with the correct surgical field to prevent damage to these structures.

## 1 INTRODUCTION

Laparoscopic cholecystectomy is a very common surgery. Anatomical variations of the biliary tree system are frequently observed findings. The incidence of extrahepatic bile duct anatomical variations was reported to be as high as 47%.^1^ Among them, the aberrant subvesical bile duct is a peculiar and rare anatomical structure around the gallbladder (GB) bed and subhepatic region, with a prevalence of 4%.^2^ Surgeons should always be careful because bile leakage is inevitable if such dim structures are damaged during cholecystectomy. We describe the case of a patient with bile leakage due to injury of an aberrant subvesical bile duct after laparoscopic cholecystectomy who underwent a relaparoscopy and biliary endoscopy.

## 2 CASE REPORT

### 2.1 Case history/examination

A 78‐year‐old man visited our emergency room with unconsciousness. According to his family, he was poisoned with pesticide (glyphosate) for self‐injury purpose. Two years ago, he had a history of cerebral infarction, and he was taking aspirin and medication for hypertension. During gastric lavage in the local clinic emergency room, his heart rate and respiration decreased. Then, he was transferred to the intensive care unit after intubation.

### 2.2 Differential diagnosis, investigations, and treatment

Intensive care such as continuous renal replacement therapy and mechanical ventilation was performed in the intensive care unit, and colistin and minocycline were administered for pneumonia. Pulmonary thromboembolism was confirmed by chest computed tomography (CT), and anticoagulant therapy was performed. Acute acalculous cholecystitis occurred simultaneously, and a percutaneous transhepatic GB drainage tube was inserted.

After 2 months of intensive treatment, the patient's condition improved, and cholecystectomy was planned. He underwent laparoscopic cholecystectomy, and there were no remarkable details of the surgery.

**Figure 1:**
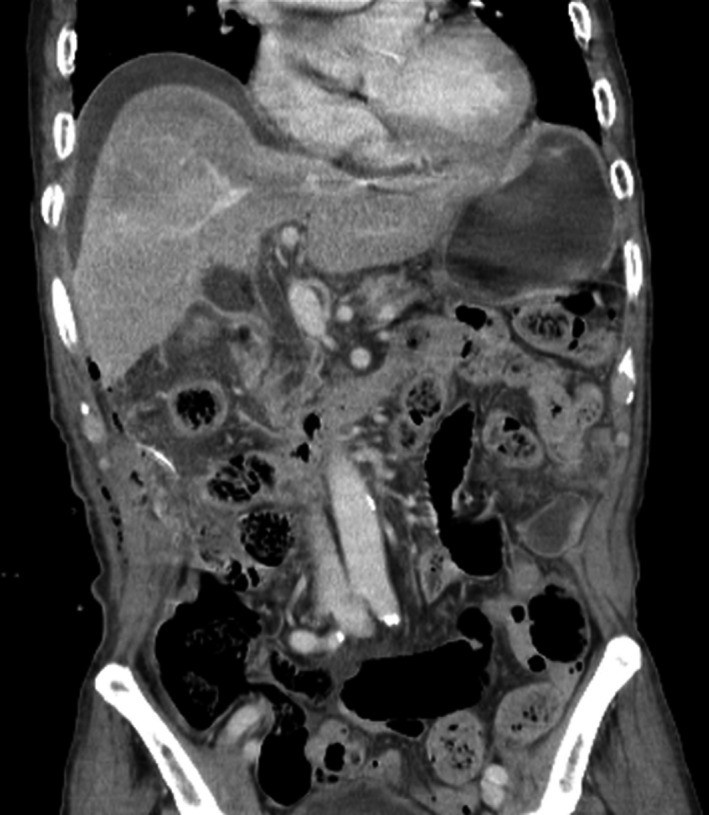
Computed Tomography (CT) Scan after Bile Leakage. CT shows pneumoperitoneum in the upper abdomen and diffuses fluid collection in the right perihepatic space

On the first day postoperatively, the drainage changed to bile. The patient's vital signs were as follows: blood pressure, 160/105 mm Hg; pulse rate, 143 beats/min; respiratory rate, 37 breaths/min; and body temperature, 39°C. There was tenderness and rebound tenderness in the whole abdomen, and CT showed pneumoperitoneum and diffuse fluid collection in the right perihepatic space (Figure 1). We diagnosed him as having bile peritonitis, and reoperation was decided.

**Figure 2:**
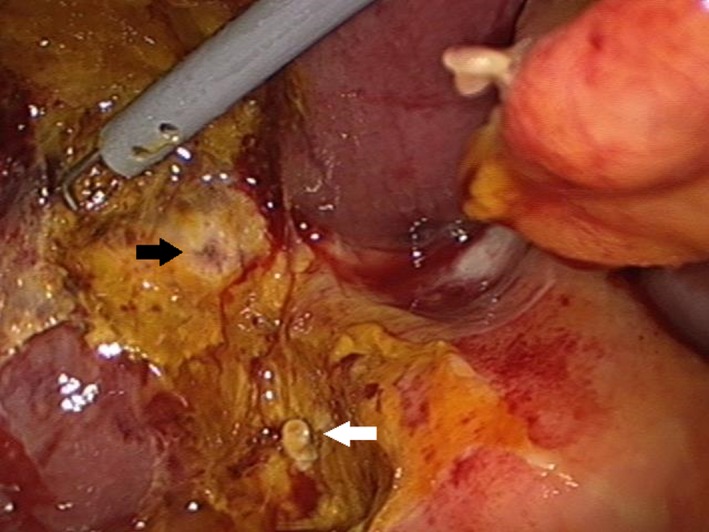
Photograph of the Surgical Field During Relaparoscopy. The bile leakage is seen in the gallbladder bed (black arrow), and it is thought to be an aberrant subvesical bile duct. The white arrow is the cystic duct stump, and there is no bile leakage at the site

On relaparoscopic examination, there was a large amount of bile juice around the GB bed and perihepatic space, and suction and irrigation were performed. The cystic duct stump was confirmed, but there was no leakage. In the GB bed, a leak was observed in a small duct‐like structure, which was regarded as an aberrant duct, and the operation was terminated after primary repair (Figure 2).

### 2.3 Outcome and follow‐up

Until 6 days postoperatively, hemoserous fluid was drained from the drainage tube. However, from postoperative day 7, the fluid changed to bile, and a percutaneous drainage (PCD) catheter was inserted into the GB bed area after performing CT.

**Figure 3:**
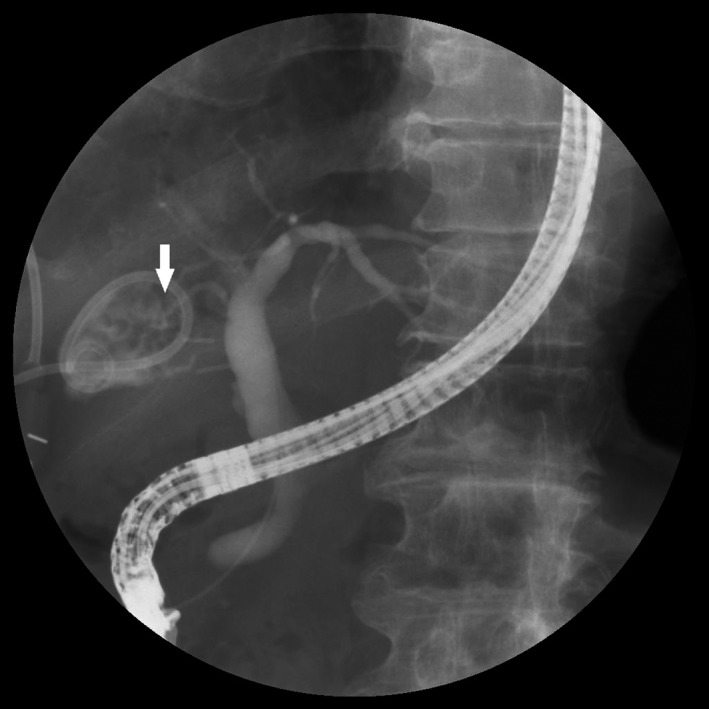
Fluoroscopic Image of Endoscopic Retrograde Cholangiopancreatography and Endoscopic Sphincterotomy. It is confirmed that the contrast is leaking from the branch of the right posterior hepatic duct (white arrow)

**Figure 4:**
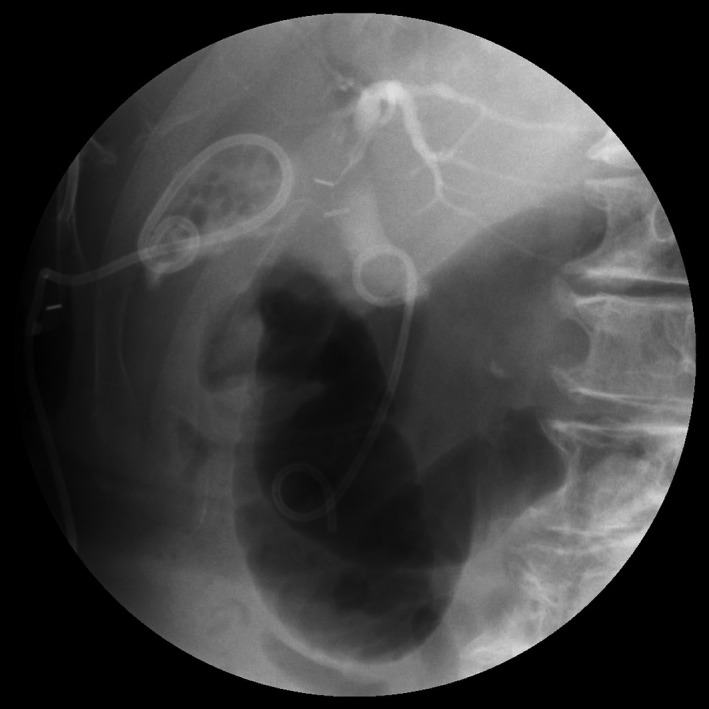
Fluoroscopic Image of Insertion of the Endoscopic Retrograde Biliary Drainage (ERBD) Stent. The ERBD stent (7 French, 5 cm) is traversed across the common bile duct

On postoperative day 9, endoscopic retrograde cholangiopancreatography (ERCP) was performed, and a leak was detected in the branch of the right posterior hepatic duct, which was regarded as an aberrant subvesical bile duct (Figure 3). Endoscopic sphincterotomy (EST) and insertion of an endoscopic retrograde biliary drainage (ERBD) stent (7 French, 5 cm) were performed (Figure 4).

### 2.4 Ten days later, the PCD catheter was removed, and he was discharged

**Figure 5:**
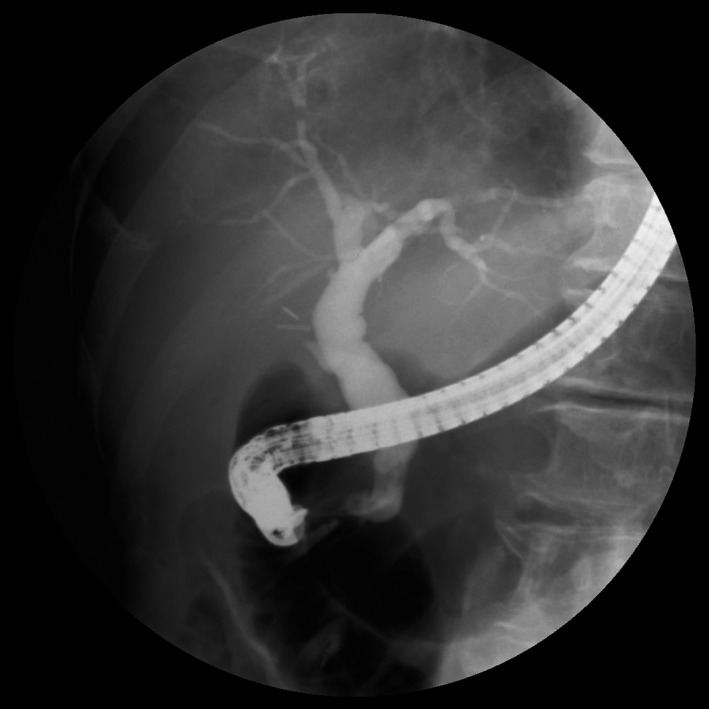
Fluoroscopic Image of Follow‐up Endoscopic Retrograde Cholangiopancreatography (ERCP). ERCP is performed again and confirms no more bile leakage

One month after ERBD stent insertion, ERCP was performed again to confirm that there was no leak, and then, the ERBD stent was removed (Figure 5).

## 3 DISCUSSION

The subvesical bile duct, commonly referred to as the duct of Luschka, is a rare anatomic variation of the biliary system across the GB fossa.^2^ The duct of Luschka is also called an accessory biliary duct, *vasa aberrantia*, subvesicular duct, or supravesicular duct.^3^ Although the exact definition and anatomical classification are not uniform until now, Schnelldorfer et al^2^ classified subvesical bile ducts into four types: (a) segmental or sectorial, (b) accessory, (c) hepaticocholecystic, and (d) aberrant bile ducts.

Aberrant subvesical bile ducts consist of biliary networks within the connective tissue of the GB fossa and usually have an average diameter of 2 mm (range 1‐18 mm).^2^ The actual morbidity rate associated with the aberrant subvesical bile duct is still unclear.^2^ Because of its small diameter, it is very difficult to confirm its presence with preoperative imaging. The reason for the clinical significance of such aberrant subvesical bile ducts is that biliary complications such as bile leakage can occur when this faint structure is damaged. Recent studies have suggested that bile leakage after cholecystectomy occurs in about 0.4%‐1.2% of cases.^4^ In fact, about 27% of this apparent bile leakage is caused by subvesical bile duct injury.^2^ If bile leakage is confirmed visually during surgery, it can be solved by simple ligation. However, if bile leakage occurs postoperatively, bile can be drained from the drainage tube after surgery; symptoms such as fever, chills, and abdominal pain may occur. If the amount of bile leakage is small, it is regarded as minor leakage, and most surgeons wait until it resolves by itself. However, if the bile leak does not resolve by itself, conventional endoscopic treatment should be performed.^5^ When ERCP is performed and a bile leak is detected in the aberrant subvesical bile duct, EST and insertion of an ERBD stent are usually considered. The purpose of this treatment is to lower the pressure in the bile ducts and to wait the spontaneous recovery. It usually takes 3‐12 days for the bile leakage to disappear after endoscopic treatment.^6^ Some studies have shown that some patients who underwent relaparoscopy selectively had a shorter hospital stay than those who underwent ERCP as the intervention.^7^

In rare cases, bile leakage can lead to life‐threatening peritonitis. Herein, the patient had a peritonitis symptom immediately postoperatively. He underwent relaparoscopy for simple suture ligation of the bile leakage site, but the bile leakage recurred and eventually resolved by ERCP.

In summary, aberrant subvesical bile ducts are very rare and may not be well identified during hepatobiliary surgery. Damage to these ducts leads to bile leakage and can result in life‐threatening complications. Therefore, surgeons who perform laparoscopic cholecystectomy should be cautious that such a structure may be present, and surgery should be performed with the correct surgical field to prevent damage to these structures.

## AUTHORSHIP

All authors were involved in patient surgery and care. S. Song and S. Jo: drafted the manuscript. All authors reviewed and approved the final draft for publication.

## CONFLICT OF INTEREST

None declared.
